# *Belminus santosmalletae* (Hemiptera: Heteroptera: Reduviidae): New Species from Panama, with an Updated Key for *Belminus* Stål, 1859 Species

**DOI:** 10.3390/insects12080686

**Published:** 2021-07-30

**Authors:** Carolina Dale, Silvia Andrade Justi, Cleber Galvão

**Affiliations:** 1 Laboratório de Biodiversidade Entomológica, Instituto Oswaldo Cruz, Fiocruz, Av. Brasil 4365, Rio de Janeiro 21040-900, Brazil; dale@ioc.fiocruz.br; 2 The Walter Reed Biosystematics Unit, Smithsonian Institution Museum Support Center, Suitland, MD 20746, USA; silvinhajusti@gmail.com; 3 Entomology Branch, Walter Reed Army Institute of Research, Silver Spring, MD 20910, USA; 4 Department of Entomology, Smithsonian Institution National Museum of Natural History, Washington, DC 20560, USA; 5 Laboratório Nacional e Internacional de Referência em Taxonomia de Triatomíneos, Instituto Oswaldo Cruz, Fiocruz, Av. Brasil 4365, Rio de Janeiro 21040-900, Brazil

**Keywords:** vector, Chagas’ disease, taxonomy, Santa Fé district

## Abstract

**Simple Summary:**

A new species of *Belminus,* discovered during the study of unidentified Triatominae specimens from the Hemiptera collection of the National Museum of Natural History, Smithsonian Institution, Washington DC, USA is described here. After comparison with previously described species, significant morphological and morphometric differences were observed, confirming the discovery of a new triatomine species, *Belminus santosmalletae*.

**Abstract:**

*Belminus santosmalletae*, a new triatomine species, is described based on a specimen from Panama, deposited in the collection of the National Museum of Natural History (NMNH), Smithsonian Institution, Washington, DC, USA. Attempts failed to identify this specimen using the keys by Lent and Wygodzinsky (1979) and Sandoval et al. (2007). A comparison was made with specimens of *Belminus* Stål, 1859 specimens deposited at the Triatominae collection at the Oswaldo Cruz Institute (CTIOC), Rio de Janeiro, Brazil; and with previous descriptions of *Belminus* species. These comparisons showed the specimen represents a new species, described in the present paper. It differs from other species of the genus mainly by the grainy tegument, scarce pilosity along the body, and the number of tubercles observed on the pronotum.

## 1. Introduction

**Figure 1:**
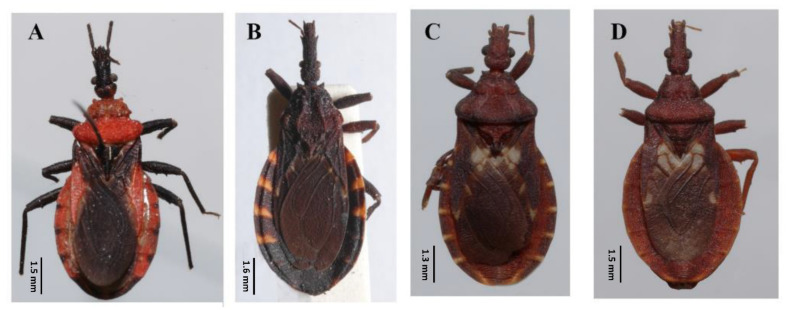
Tribe Bolboderini genera: (**A**) *Bolbodera* Valdés, 1910 (female specimen of *Bolbodera scabrosa* Valdés, 1910); (**B**) *Belminus* Stål, 1859 (female specimen of *Belminus peruvianus* Herrer, Lent and Wygodzinsky, 1954); (**C**) *Microtriatoma* Prosen and Martínez, 1952 (male specimen of *Microtriatoma borbai* Lent and Wygodzinsky, 1979); (**D**) *Parabelminus* Lent, 1943 (male specimen of *Parabelminus carioca* Lent, 1943). All specimens are deposited in the Triatominae collection (CTIOC), Oswaldo Cruz Institute, Fiocruz, Brasil.

The subfamily Triatominae (Hemiptera: Reduviidae) comprises 153 extant and three fossil species assigned to five tribes [1,2,3,4] with all the extant species being considered potential Chagas disease vectors. The tribe Bolboderini has been considered a monophyletic group, and includes the genera *Bolbodera* Valdés, 1910; *Belminus* Stål, 1859; *Parabelminus* Lent, 1943; *Microtriatoma* Prosen and Martínez, 1952 [4] (Figure 1).

**Figure 2:**
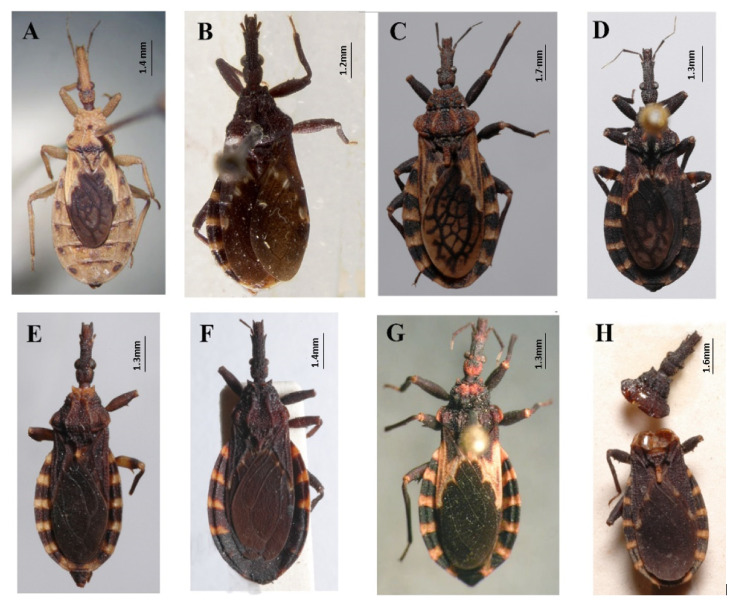
Eight described species of *Belminus* genus: (**A**) *Belminus corredori* Galvão and Angulo 2006 (male, holotype deposited in Triatominae collection, Instituto Oswaldo Cruz (CTIOC)); (**B**) *Belminus costaricensis* Herrer, Lent and Wygodzinsky, 1954 (male, holotype deposited in Smithsonian Museum (NMNH), photographed by Thomas Henry); (**C**) *Belminus ferroae* Sandoval, Pabón, Jurberg and Galvão, 2007 (male, holotype deposited in CTIOC); (**D**) *Belminus herreri* Lent and Wygodzinsky, 1979 (female deposited in CTIOC); (**E**) *Belminus laportei* Lent, Jurberg and Carcavallo, 1995 (female paratype deposited in CTIOC); (**F**) *Belminus peruvianus* Herrer, Lent and Wygodzinsky, 1954 (female paratype deposited in CTIOC); (**G**) *Belminus pittieri* Osuna and Ayala, 1993 (female, holotype deposited in Museo del Instituto de Zoología Agrícola (MIZA), photographed by Marco Giani in Osuna and Ayala, 1993); (**H**) *Belminus rugulosus* Stål, 1859 (male deposited in CTIOC).

The genus *Belminus* was described by Stål [5] based on a single species, *Belminus rugulosus*, from Colombia [6]. The group is well characterized and can be easily differentiated from other triatomines by the small total body length (8.5–12 mm); elongate and fusiform head; dorsoventrally compressed labium, with the first and second visible segments elongate and subequal in length and longer than the third segment; the presence of 6–7 small discal tubercles on the anterior lobe of pronotum and connexivum with a dorsal longitudinal ridge [7,8]. It is the most diverse genus of Bolboderini, with eight previously described valid species: *Belminus corredori* Galvão and Angulo, 2006; *Belminus costaricensis* Herrer, Lent and Wygodzinsky, 1954; *Belminus ferroae* Sandoval, Pabon, Jurberg and Galvão, 2007; *Belminus herreri* Lent and Wygodzinsky, 1979; *Belminus laportei* Lent, Jurberg and Carcavallo, 1995; *Belminus peruvianus* Herrer Lent and Wygodzinsky, 1954; *Belminus pittieri* Osuna and Ayala, 1993; *Belminus rugulosus* Stål, 1859 [9] (Figure 2A–H).

**Figure 3:**
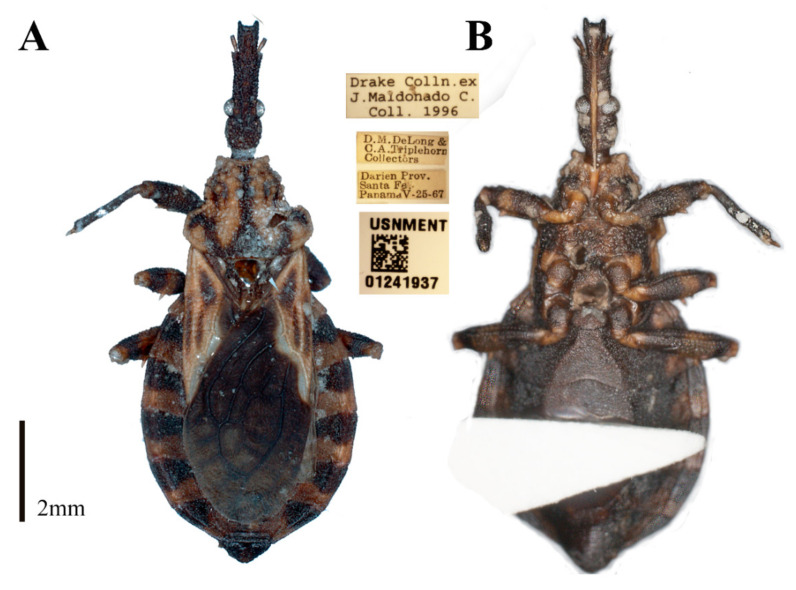
*Belminussantosmalletae* sp. n. female (**A**) dorsal view and (**B**) ventral view, and three labels with information about collection, collectors, date, local, and deposit voucher (USNMENT01241937).

During a study of the Hemiptera collection at National Museum of Natural History, Smithsonian Institution (NMNH), Smithsonian Institution, Washington DC, USA, a specimen belonging to the genus *Belminus* was found. Upon comparison with other specimens of the genus deposited in the Triatominae collection of the Oswaldo Cruz Institute (CTIOC), and with the descriptions of the other *Belminus* species, it was clear that such specimen represents a new species. Here, we describe *Belminus santosmalletae* sp. n. (Figure 3) based on this single female specimen.

## 2. Materials and Methods

Measurements and observations were made using a Dino-Lite Edge digital microscope. Dorsal habitus images and detailed photos were taken using the wide zoom microscope OlympusDSX100 camera. The following characters and terminology used for description based on Lent and Wygodzinsky [8] and Sandoval et al. [9].

## 3. Results

### 3.1. Taxonomy

Family Reduviidae Latreille, 1807

Subfamily Triatominae Jeannel, 1919

Tribe Bolboderini Usinger, 1944

Genus *Belminus* Stål, 1859

Type species *Belminus rugulosus* Stål, 1859

**Figure 4:**
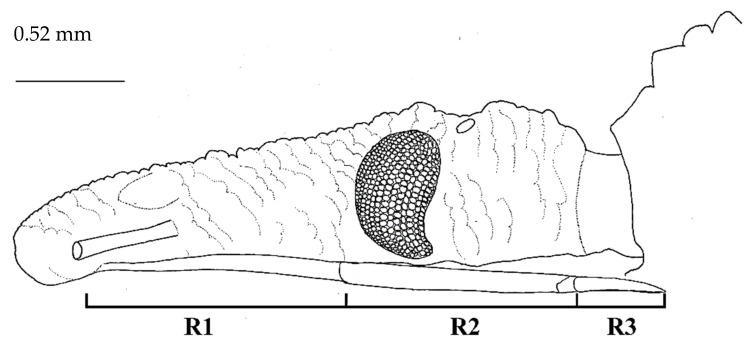
*Belminus santosmalletae* sp. n. Schematic outline of the detail of the head in lateral view showing the visible labium segments (**R1**, **R2**, and **R3**), depicting the first segment (**R1**) not reaching the anterior border of the eyes.

**Figure 5:**
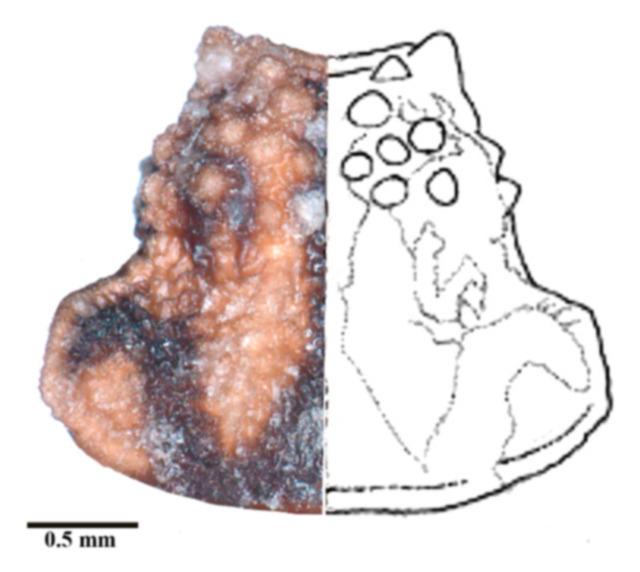
Dorsal view of pronotum photograph (right side) and schematic outline (left side) of *Belminus santosmalletae* sp. n., showing the anterior lobe with 7 + 7 discal tubercles and 2 + 2 lateral tubercles.

**Figure 6:**
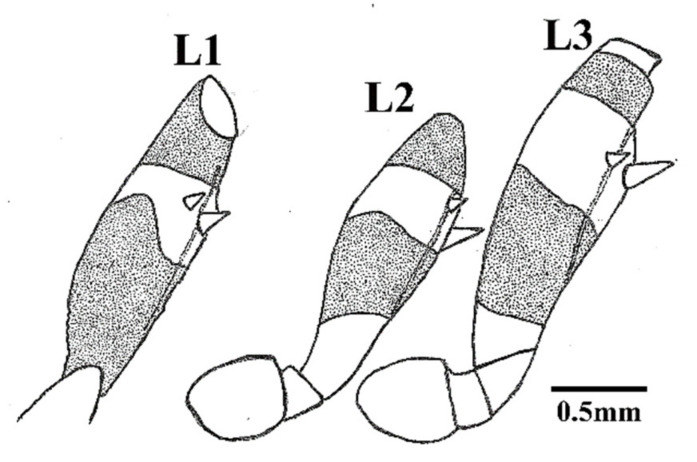
Schematic outline of the legs of *Belminus santosmalletae* sp. n., showing the denticles (**L1**—fore; **L2**—middle; **L3**—hind leg).

**Figure 7:**
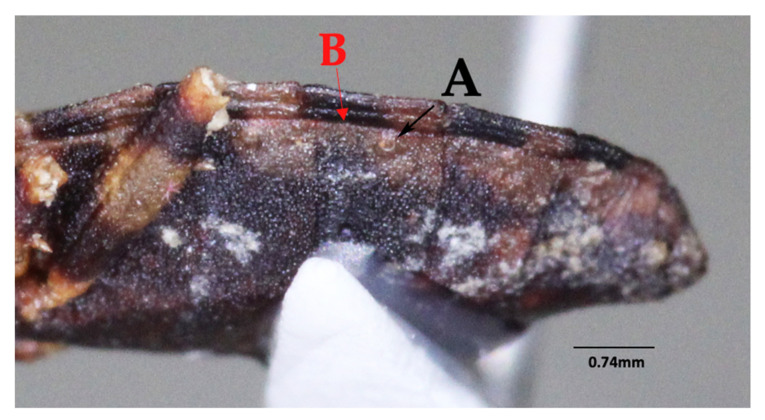
Detail of abdomen of *Belminus santosmalletae* sp. n., depicting the spiracles (**pointed by arrow A**) being very close but not adjacent to the connexival suture (**pointed by arrow B**).

*Belminus santosmalletae* sp. n. Dale, Justi and Galvão

(Figure 3A,B, Figure 4, Figure 5 and Figure 6 and Figure 7A,B)

Type material. Holotype: female, PANAMA, Darién Province: Santa Fé District, 25 May 1967. USNMENT01241937, deposited in the Hemiptera Collection of the National Museum of Natural History, Smithsonian Institution, Washington DC 20002, USA.

### 3.2. Description

#### 3.2.1. Coloration

Overall color brown to yellowish-brown, with light and dark brown areas on the pronotum, hemelytra, connexivum, legs, and abdomen; with a few spots paler than the integument. Visible labial segments brown to yellowish-brown. Pronotum predominantly yellow with brown stripes and spots. Scutellum brown with the apex of scutellar process yellow. Femora with a subapical yellow ring. Corium yellow with three dark brown areas, one external, almost straight, the other internal. Membrane of hemelytron dark brown, veins darker with secondary venation within cells visible but not conspicuous. Connexival segments with transverse marks, anterior dark brown marks wider than posterior dark yellow marks (Figure 3A,B).

#### 3.2.2. Morphological Features

Female. Total length 11.45 mm, width of pronotum 2.75 mm, and width of abdomen 4.63 mm. The integument of the entire body was very grainy with short pilosity, except for the hemelytra.

Head. Elongated, fusiform, very granulose, three times as long as wide (1:0.31), slightly longer than pronotum (1:0.84). Clypeus truncated on the apex. The anteocular region was more than twice as long as postocular region (1:0.41). The postocular region was sub-circular, with sides which were convex and convergent posteriorly. The genae was compressed laterally, with the apices, considerably, surpassing anteclypeus. The external spinelike projection of the antenniferous tubercle was short, and barely extending beyond the base of the first antennal segment. Antennae were inserted apically, a third of the way through the anteocular, towards the postocular region. Antennal segments were missing, except in segment I. Eyes in the lateral view reached the level of the lower but not the upper surface of the head (Figure 4). The ratio between eye width and synthlipsis was 1:2.1. Ocelli were very small, not elevated, laterally oriented, and situated at the level of the integument. The visible labial segments of the first segment was longer than second, not reaching the anterior border of the eyes, while the third visible segment barely reached the anterior portion of the stridulatory sulcus; the length ratio of visible labial segments was 1:0.8:0.4 (Figure 4).

Pronotum. The width of the pronotum was slightly bigger than the length (1:0.83). The anterior lobe was narrow, granulose, with 7 + 7 conspicuous discal tubercles and 2 + 2 conspicuous lateral tubercles (Figure 5), sides forming a conspicuous angle at the junction of the posterior lobe sides. The anterolateral processes were short, subtriangular, and rounded apically. Th posterior lobe was granulose with submedian carinae almost attaining the posterior border of the pronotum. The scutellum was triangular, with the apex of the scutellar process being long, subcylindrical, and not pointed apically. Further scutellum characters are not available for this specimen, as it was primarily pinned through the scutellum. Prosternum with 1 + 1 projections, lateral to the stridulatory sulcus.

Legs. Short and stout. Femora were sulcate at the venter, and with 2 + 2 subapical denticles, one being distinctively larger (Figure 6). Tibiae were slender and compressed laterally with short pilosity. The specimen was without tarsi.

The hemelytra fell distinctly short of the apex of abdomen, with venations consistent with the genus description. Abdomen. Abdominal venter was flattened, and the connexivum was wide dorsally with a dorsal ridge. The spiracles were close but not adjacent to the connexival suture (Figure 7).

**Remarks on the conditions of the specimen.** The specimen was found without the last three antennal segments, without tarsi, and with scutellum damaged by the pin.

Diagnosis. According to the comparative morphology of the genus, *Belminus*, *B. santosmalletae*, it seems to be closer to the species *B. ferroae* and *B. pittieri*, and can be distinguished by the yellow rings on all femora (these rings can be found in *B. herreri* and *B. laportei*, but the morphology of the wing is distinct), grainer integument, scarce pilosity, lighter and larger yellow spots on the pronotum, 7 + 7 discal tubercles on the pronotum, rather than 6 + 6 and darker membrane, with little color contrast between the cells and veins.

### 3.3. Etymology

This species is dedicated to Dr. Jacenir dos Santos-Mallet, a Brazilian researcher at the Fundação Oswaldo Cruz, in Rio de Janeiro, Brazil. She has dedicated over 30 years to the research of tropical disease vectors.

### 3.4. Key to the Species of Belminus

**1.**-Overall color light orange, brachypterous
***B. corredori***


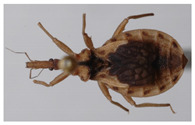

-Overall color predominantly brown or black, macropterous2

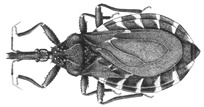

**2.**-Pronotum totally dark3

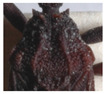

-Pronotum with a yellow or orange pattern5

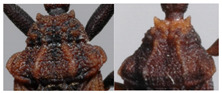

**3.**-First visible labial segment surpassing the anterior border of the eye in lateral view; scutellar process compressed dorsoventrally with a conspicuous dorsal sulcus
***B. costaricensis***


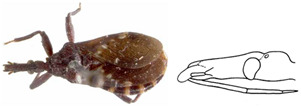

-First visible labial segment not surpassing the anterior border of the eye in lateral view. Scutellar process not compressed, with or without a sulcus4

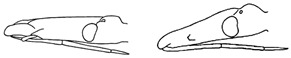

**4.**-First visible labial segment longer than the second, its apex almost attaining the anterior border of eye in lateral view. Postocular region of head with sides distinctly convex. Scutellar process yellowish or orange, subcylindrical with a narrow longitudinal sulcus 
***B. rugulosus***


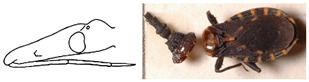

-First visible labial segment not longer than second, its apex far from the anterior border of the eye in lateral view; Scutellar apical process black conical without sulcus
***B. peruvianus***


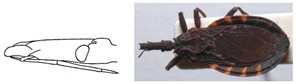

**5.-**Corium almost totally black6

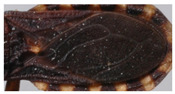

-Corium light colored7

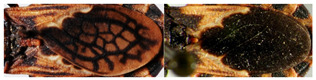

**6.**-Pronotum with posterior and lateral border light colored. Scutellar process not sulcate. Membrane of hemelytron with base light colored. Abdomen mainly dark connexivum with yellow spots, extending to the border of sternites. Hind femora with a yellow ring
***B. herreri***


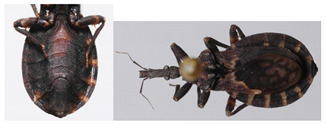

-Pronotum without posterior and lateral border light colored. Scutellar process sulcate. Membrane of hemelytron dark. Ventral area of abdomen mainly yellow with black stripes. Hind femora yellow in central two-thirds
***B. laportei***


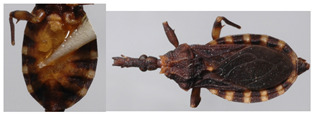

**7.**-Legs black with an orange-red color at the apex of each femur and bases of tibiae8

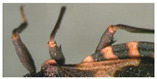

-Legs black with a yellow ring on all femora
***B. santosmalletae* sp. n.**


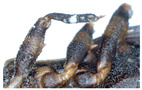

8.-Overall color black, with orange areas on connexivum and corium. Hemelytra membrane black, without reticular cells, color of veins not contrasting with cells
***B. pittieri***


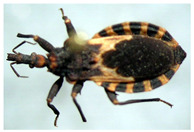

-Overall color dark brown, with several orange or yellowish areas through the body. Hemelytral membrane light brown contrasting with the dark veins. Cells reticulate 
***B. ferroae***


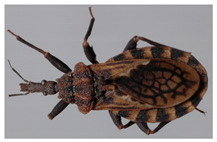



## 4. Discussion and Conclusions

The genus *Belminus* currently comprises nine valid species, with distribution ranging through Central America, Colombia, Peru, Venezuela, and northern Brazil. The genus, previously thought to be exclusively sylvatic (arboreal), was recently found inside human dwellings in some locations in Colombia and Peru [10]. To this date, however, there has been no report of infection by Trypanosoma cruzi (Chagas 1909), the etiological agent of Chagas disease.

A unique feature within Triatominae is also the fact that *Belminus* species have been found feeding on other insects, especially cockroaches, showing a broader host preference. Wheeler and Pennak [11] considered *Belminus corredori* one of the 100 most interesting species discovered in this century, and according to Gil-Santana [4] this fact launched “an unprecedented focus on the uniqueness of the representatives of this tribe”.

*Belminus santosmalletae* is the ninth species described to this understudied genus, and the second from Panama. In addition to the original descriptions [8,12,13], taxonomic knowledge on the genus is restricted to a few morphological papers reflecting the fact that this is the one of the least known triatomine genera. Herrer et al. [7] made the first descriptions of eggs and nymphs of first and fifth instars of *B. peruvianus*, and only after 31 years, Rocha et al. [14] described all instars of one *Belminus* species: *B. herreri*. They provide a complete description, as well as ontogenetic morphometrics of post-embryonic head development. Gil-Santana and Galvão [15] described the male genitalia of *B. rugulosus* and *B. corredori* and provided a discussion on previous data in the literature on male genitalia of other species. It is impossible to make inferences on the biology of *B. santosmalletae* because, like *B. pittieri,* the description is based on a single female specimen. Additionally, a search on GenBank shows a single sequence (GenBank access AJ421964) from this group, highlighting the lack of data available to further studies on integrative taxonomic approaches for the genus.

## Data Availability

Not applicable.
